# Difficulties and Problem-Solving Strategies in Wayfinding Among Adults With Cognitive Disabilities: A Look at the Bigger Picture

**DOI:** 10.3389/fnhum.2020.00046

**Published:** 2020-02-18

**Authors:** Romain Delgrange, Jean-Marie Burkhardt, Valérie Gyselinck

**Affiliations:** ^1^LAPEA, Univ. Gustave Eiffel, IFSTTAR, Versailles, France; ^2^Université de Paris, LAPEA, Boulogne-Billancourt, France

**Keywords:** spatial cognition, mobility, semi-structured interview, critical incident technique, navigational aids

## Abstract

Many people with cognitive disabilities avoid outside activities, apparently for fear of getting lost. However, little is known about the nature of the difficulties encountered and the ways in which these individuals deal with them. None of the few studies on wayfinding by people with cognitive disabilities have explored the various specific difficulties they meet in everyday life. Using both a qualitative and quantitative methodology, this study aimed at profiling the types of difficulties encountered in urban mobility and the associated problem-solving strategies. In order to provide more direct evidence from the field, we conducted semi-structured interviews using the critical incident technique (Flanagan, 1954). Among the 66 participants interviewed, 44 had cognitive disabilities and 22 were matched controls. The analysis of the transcripts showed in particular an overall reduced autonomy in problem-solving strategies for people with a cognitive disability. The multiple correspondence analysis highlighted three main types of complex situations, covering a comprehensive range of complex situations that are met in everyday life by these individuals. Results also indicated that people with cognitive disabilities request assistance from another person more frequently when a complex event occurs. These situations are discussed as potential cues for improvements in navigational aids. Conclusions and perspectives are provided to improve wayfinding among people with cognitive disabilities.

## Introduction

### Wayfinding as a Cognitive Process

Getting around the city is the first step in many of our daily activities, whether they are related to work or leisure. This activity is therefore fundamental for autonomy as well as for social integration and community access (Doig et al., 2001; Sohlberg et al., 2007). Still, finding one’s way in the environment involves more than just movement (Montello, 2017). Apart from controlled locomotion, spatial navigation relies on a set of cognitive processes referred to as “wayfinding” (Montello, 2005, 2017; Wiener et al., 2009). In his early work on urban architecture, Lynch (1960) coined the term wayfinding to describe the use of environmental cues in order to move toward a destination, considering the physical properties of cities that allow a traveler to find their way. During the following two decades, the scope of research expanded toward a more cognitive perspective, centered on human processes rather than on the environment. Wayfinding has been defined as the cognitive process of finding and following a path that links an origin to a destination (Golledge, 1992). It is considered as a spatial problem solving that depends on the construction of mental models, consisting in internal representations of distinct scales of the environment, from a landmark, first-person perspective, to a comprehensive, “bird’s-eye” view (Tolman, 1948; Siegel and White, 1975; Johnson-Laird, 1980). The use of these representations along an itinerary relies heavily on mnesic and executive functions, in order to retain spatial information and perform the adequate actions that govern movement (Vandenberg, 2016; Meneghetti et al., 2017). It involves four main cognitive components (Vandenberg, 2016): decision making, orientation, path integration, and closure. The first step, decision-making, implies that several factors have been taken into account, such as selecting the adequate path between the origin and the destination of the trip (Gärling et al., 1986; Golledge, 1995). Decision making also takes place during the trip: while planning and moving through an itinerary, people use their internal representations of the environment to automatically choose and follow a path (Richter, 2007; Brunyé et al., 2010). A second cognitive resource that supports the use of mental models to find one’s way is orientation, the capability of knowing where an individual finds themselves in the environment, in relation to the surroundings (Vandenberg, 2016). A third process deals with updating orientation while moving through the environment, keeping track of the motion and continually acquiring information on the environment to maintain the knowledge of one’s location in space (Gärling et al., 1986). Finally, the last step and fourth component is closure (Vandenberg, 2016), i.e., realizing that one has reached the intended destination.

Considering the central role of cognition in wayfinding, any condition that affects either internal spatial representations or cognitive processes can result in difficulties in finding one’s way (Postma and van der Ham, 2016). Depending on how challenging the environment is (e.g., noisy or dark), one’s current state of health, level of fatigue or stress, nobody is “permanently unimpaired” (Arthur and Passini, 2002). This becomes even truer in the case of permanent cognitive disabilities resulting from strokes or head injuries.

### What We Know About Wayfinding in People With Cognitive Disabilities

For a long time, neuropsychology has documented difficulties in spatial representations resulting from cognitive disabilities. These studies make clear the impairments as well as their neuroanatomical correlates, and classify several types of disorientation (for reviews, see Aguirre and D’Esposito, 1999; Claessen and van der Ham, 2017). Studies in cognitive psychology have also highlighted the importance of working memory in spatial representations using interference paradigms (Gyselinck et al., 2009) and have shown how cognitive aging could impair these processes (Meneghetti et al., 2012).

Only a few studies gathered evidence of specific difficulties in wayfinding among people with cognitive disabilities, based on both interviews and experimental settings. Results showed that people with cognitive disabilities appear to lack the ability to link landmarks and paths in a bird’s-eye view of their everyday environment (Antonakos, 2004), and that they show little independence when facing a complex situation (Lemoncello et al., 2010). In particular, when observing problem-solving situations, Lemoncello et al. (2010) showed that people without cognitive disabilities mostly resolve spatial problems independently by either guessing or walking a little further to look for a landmark. Conversely, people with cognitive disabilities ask the accompanying experimenter for help, or suggest potential solutions that are generally judged to be vague by the experimenter. These findings seem consistent with these individuals’ lifestyle: based on group interviews, Sohlberg et al. (2005) showed that they avoid going outside for fear of getting lost, restricting themselves mostly to routine outside trips.

Up to now, the characteristics of the difficulties encountered by people with cognitive disabilities when traveling outside in their daily activities have remained largely unexplored (Meissonnier, 2016; van der Ham and Claessen, 2016; Nakamura and Ooie, 2017). While “getting lost” appears to be a major factor of avoidance of getting around the city (Sohlberg et al., 2005), one can only conjecture on the nature of this problem, its causes and consequences, and the specificity these characteristics represent for the target population in comparison to the general public. Moreover, not much is known about the problem-solving strategies these people actually implement in their everyday life when they face complex situations, whether these consist in getting lost or not.

The scarcity of research data on this topic seems to be caused by a difficulty in recruiting and categorizing participants with cognitive disabilities, some matters directly discussed by most authors (Dawson and Chipman, 1995; Sohlberg et al., 2005, 2007; Lloyd et al., 2009; Cho et al., 2017). For the last 40 years, very few navigational aids have been designed to meet human spatial cognition needs and functioning (Grison and Gyselinck, 2019). This is even truer for people with cognitive disabilities (Sohlberg et al., 2005, 2007), in part due to the lack of information on the nature of the difficulties encountered by the target population in everyday life.

### Objectives of the Study

The present study was designed to explore representative everyday situations in order to develop a broader understanding of the effects that cognitive disabilities can have on all the components involved in completing an itinerary. This means not only taking the right decision at a crossroads but also coping with an unexpected delay in transport, getting along with other pedestrians or simply recognizing a building as the destination of the trip (Vandenberg, 2016). We therefore collected the existing experience of complex wayfinding events among people with and without cognitive disabilities. In particular, we explored the problem-solving strategies used when a complex or an unexpected event occurs. We address the potential specificity of the difficulties met by people with cognitive disabilities by comparing them with a matched control group. Based on these results, we provide some insights that should prove helpful in designing better adapted navigational aids. Our results could also clarify the features of ecological wayfinding situations experienced as complex by people with disabilities, opening up avenues for future research.

Note that the difficulty for people with cognitive disabilities in recalling and articulating specific experiences and feelings (Paterson and Scott-Findlay, 2002) usually prevents the use of semi-directed interviews. Still, the documented benefits, such as avoidance of bias and facilitation in user participation, have led some researchers to advocate such investigations, provided certain precautions are taken (Heal and Sigelman, 1995; Cambridge and Forrester-Jones, 2003; Gilbert, 2004). Moreover, results have shown that interviewing the relatives of these people is not sufficiently reliable when investigating outside activities, suggesting that the target population itself should be included rather than proxies (Cusick et al., 2000).

Thus, to address our research questions, individual semi-directed interviews were conducted based on the “critical incident technique” (Flanagan, 1954; see section “Materials and Methods”) in order to perform a step-by-step exploration of representative, detailed everyday-life wayfinding experiences. This technique allows the problematic aspects of complex situations to be rapidly highlighted and offers a way to investigate activities that would otherwise be difficult to observe in laboratory settings. Initially developed to gather data with task experts in order to identify critical competencies for their job, the aim of the technique is to avoid the collection of general thoughts and stereotypes about a theme and to favor verbal reports on specific experienced situations recognized by participants to be significant for the theme under investigation. The features of the critical incident technique make it particularly relevant for the study of complex situations such as getting around a city, and for urban mobility in general (Corneloup and Burkhardt, 2016; Grison et al., 2016). Furthermore, a questionnaire on orientation and spatial abilities (Pazzaglia et al., 2000) was administered to characterize the participants of both groups.

As to our knowledge, this is the first research study on wayfinding to use the critical incident technique with people with cognitive disabilities, we adopted an exploratory perspective. We considered every potentially complex situation that the participants recalled, whether they concerned the action of getting lost or an unpleasant trip in a crowded subway. The aim was to determine the most frequent profiles of complex situations, and whether they were associated with a group of participants or not.

## Materials and Methods

### Participant Recruitment

Two groups of participants were recruited. The experimental group was formed on the basis of the following inclusion criteria: being at least 18 years old at the time of the interview (French legal majority), presenting a legally authenticated cognitive disability, and being able to travel alone in town. Participants had to be stabilized and lived autonomously. An exclusion criterion was the existence of disabilities impacting the visual or motor functions, thereby creating difficulties in mobility possibly unrelated to the cognitive disability itself. Forty-seven volunteers with a cognitive disability and meeting our inclusion criteria came forward to participate in this study. Forty-three came from four partner institutions: 10 participants came from home care services and specialized services for handicapped adults (French “SAMSAH”), and 33 were workers in centers providing care through employment to handicapped adults (French “ESAT”). One participant came from the investigator’s indirect network. Participants from the institutions were initially identified and invited by the professionals to volunteer for the study. It was made clear to them that any participant strictly meeting the inclusion criteria could volunteer, whether they had already expressed a mobility complaint or not. With the exception of aphasia that would prevent interviewing, no additional selection criteria were applied by the professionals. Volunteers were then contacted directly by the experimenter for an appointment at their home or within the institution when possible. Three participants in the experimental group were excluded since they expressed difficulties understanding the questions during the interview, and the session was therefore interrupted.

The control group was formed based on the following inclusion criteria: being at least 18 years old at the time of the study, absence of cognitive impairment and absence of daily use of a car as a driver. The latter criterion was applied because no experimental participant declared driving. Also, the partner institutions for the experimental group were located in the outskirts of the cities, and the participants themselves often lived in the suburbs. Therefore, control participants were recruited among companies based outside the city center, and had to use different types of transport (mainly trains and buses) every day, in order to match the environmental context of the experimental group.

### Sample Characteristics

The experimental group included 44 participants (28 men, 16 women). The mean age was 38.91 years (Minimum = 21, Maximum = 81, *SD* = 13.50). Amongst them, five participants suffered from the after-effects of strokes (of whom two had had two strokes), nine from traumatic head injury, one from a brain lesion after surgical tumor removal, two were epileptic, four had developmental cognitive disabilities and 23 suffered from cognitive disabilities of unspecified etiologies.

While cognitive disabilities, like motor disabilities, can refer to a wide variety of difficulties, they cannot be specified in a “standardized” way either by a device (e.g., a wheelchair, crutches) or a functional disability (e.g., blindness). As documented in the neurological literature, there are potentially as many disabilities as lesions. More than half of the participants in our study suffered from cognitive disabilities of unspecified etiologies, suggesting pathogenesis heterogeneity. As we chose to focus on complex events in real life, we selected people who traveled autonomously for their everyday activities. Therefore, our participants were not under medical care. Furthermore, most of them did not have access to their neuropsychological and medical specifics. We therefore used the following two inclusion criteria: the stability of the disability, and the legal authentication of cognitive disabilities according to the 2005 disability policy reform. The authentication procedure consists in successive medical examinations. All partner institutions catered only for people who had strictly complied with this procedure.

The control group included 22 participants (8 men, 14 women) meeting the criteria who volunteered to participate, with a mean age of 37.45 years (Minimum = 21, Maximum = 61, *SD* = 14.39).

### Design and Procedure

The experimenter first presented the study to the participant in accordance with the content of the information letter. When all the participant’s questions had been addressed, they were asked to sign the consent form. For the participants contacted by telephone, a first call was made to present the study; the information letter and the consent form were then sent by mail. Once the consent form had been read and signed, the interview call was made.

#### Semi-Structured Interview Using the Critical Incident Technique

The semi-structured interview was based on the critical incident technique (Flanagan, 1954). A “critical incident” is a situation specific in time and geographically localized, resulting in either a satisfactory or an unsatisfactory experience for the participant. The critical incident technique consists in a recall of these events guided step-by-step by the investigator. Each interview was audio recorded and then transcribed. The direction of the interview led the participants to describe each step of the complex situation from the general proposition “*Think of a specific moment that you experienced as complex when you moved around the city during the last few months. It can be a moment that was either pleasant or unpleasant in the end.*” The questions asked by the experimenter to guide the direction of the interview focused successively on the step-by-step process of the event, on the feelings experienced by the participant, on the participant’s reactions and strategies, and on whether the participant had learnt anything from the situation (using the question “*If you were in the same situation today, would you do the same?*”).

When a description was incomplete, the investigator prompted the participant to elaborate using interview techniques (e.g., summarizations, repetitions of keywords, pauses, nods). To complete a description, the investigator also asked the participant if they would judge the situation overall positively or negatively. When the description of one situation was over, the experimenter asked if the participant could think of another complex situation by reiterating the initial general proposition. Then, a second event was detailed in the same way. When the participant declared that they could not remember any other complex event, the investigator summarized all the situations that had been previously described by the participant in order to make sure they had not forgotten anything. The interview was concluded when the participant stated that they did not remember any other complex event. The average duration of the interview was approximately 20 min (Minimum = 3, Maximum = 58, *SD* = 10).

#### Questionnaires

Two questions about the age and gender of the participant were asked. When a participant from the experimental group was willing and able to define their disability, the experimenter asked one optional question about their etiology (e.g., stroke, head injury). Then a broader question about the participant’s travel behavior was asked to induce them to summarize their daily journeys, recall the types of transportation (i.e., pedestrian, bus, subway, train, tramway, driver or passenger of a car, bike, others) and usual durations of these journeys. This open question served as an ice-breaker for the interview and a confirmation for the investigator that the criterion of autonomous travel was indeed met.

A 16-item questionnaire on orientation and spatial abilities was then completed (Pazzaglia et al., 2000). This questionnaire returns six main scores: “general spatial orientation,” “knowledge and use of compass points,” “survey representation score,” “route representation score,” “landmark representation score,” and “preference toward survey representation over the rest.” The procedure for the completion of this questionnaire was the same for both groups. The questions were formulated orally by the experimenter, who scored the participant’s answers on the scales. Since the questionnaire had not previously been tested and adapted to people with cognitive disabilities, when a participant from the experimental group expressed a misunderstanding of certain items, the questions were exemplified or reformulated with synonyms in order to be understood by the participants. The goal of these reformulations was to stay as close as possible to the original question, while adapting to the specific cognitive disabilities of the experimental group. The average duration of this questionnaire was approximately 15 min.

### Analyses

#### Collected Data

The interviews of the experimental group took place between July and November 2017. Nine participants were met at their home, 37 were met within the institutions, and one participant recruited by the indirect network of the experimenter was contacted by phone. The interviews of the control group took place between April and June 2018. Twenty-two participants were met either at their office, at their home or by telephone depending on their availability.

The audio recordings of the 66 semi-structured interviews were entirely transcribed. Two hundred and eighteen critical incidents were obtained from the verbatim, of which 126 were from the experimental group (2.9 incidents per person in average) and 92 from the control group (4.2 incidents per person in average). Situations judged as overall positive (such as an experience deemed satisfactory or a time-saving situation) accounted for 19.9% of the cases in the control group and 5.6% of the cases in the experimental group. It is worth noting that situations triggering positive emotions and overall positive situations do not always match, as situations initially resulting in negative emotions could be evaluated as pleasant by the participants in the end.

Among the 44 questionnaires on orientation and internal spatial representations (Pazzaglia et al., 2000) from the experimental participants, three contained unanswered items. Consequently, these three questionnaires were excluded from this analysis. All 22 questionnaires from the control group were fully completed.

#### Data Coding

The verbatim of all 218 critical incidents were coded using a grid with six variables to delineate the following dimensions of complex events: cause, type, consequence, emotion, problem-solving strategy, and learning from the situation. The first step in coding the critical incidents remained as close as possible to the story recounted by the participant. The six variables from this first step included between 16 and 48 modalities. In a second step these specific modalities (e.g., “event happened because of rush hour,” “event caused by the crowds of people”) were combined into broader ones (e.g., “event caused by a punctual environmental difficulty”) in order to allow analysis. Finally, we obtained six variables ranging from 7 to 12 modalities detailed in Table 1. All the variables (except “consequence”) include a modality labeled “other” comprising unique situations that did not fit any other modality.

**TABLE 1:** Variables and modalities determined from the analysis of the interviews, with examples from the verbatim, and overview of the contingency tables for each variable.

Variable	Number of modalities	Modalities	Examples from the verbatim	Number of incidents in experimental group	Number of incidents in control group	Total
Cause of the event	9	Choosing an unusual route	*“I thought why not take this way today”*	1	3	4
		Environment legibility	*“The road sign was hidden, in the air”*	14	10	24
		Initial interindividual conflict	*“I had an argument with my friend, it stressed me on my way back”*	4	1	5
		Initial request for information	*“We asked and they gave us wrong advice”*	4	0	4
		Internal cause	*“I had forgotten there was a deviation”*	11	5	16
		Not recognizing the environment	*“The place was not like I imagined it would be”*	21	9	30
		Punctual environmental difficulty	*“It was snowy”*	20	21	41
		Transport network	*“They were on strike”*	16	19	35
		Other	*“I met a friend of mine”*	5	2	7
**Total**				**96**	**70**	**166**
Event type	12	Being in an unwanted or unusual route	*“I had to change and take another line”*	2	8	10
		Being lost	*“I couldn’t find the street”*	40	14	54
		Conflict with another person	*“There were drunk people in the station”*	9	1	10
		Disruption of the transport network	*“The subway was not working”*	25	31	56
		Harmful situation	*“I slipped on the wet manhole cover”*	5	0	5
		Missing the transportation	*“I missed the last subway”*	4	2	6
		Mistake by another person	*“The driver did not know the route”*	1	5	6
		Obstacle on the route	*“The exit we wanted to use was closed”*	3	8	11
		Physical or emotional discomfort	*“It was too hot in the train”*	6	5	11
		Route mistake	*“I took the bus in the opposite direction”*	18	11	29
		Transport not on time	*“The bus was late”*	5	1	6
		Other	*“My friend gave me a ride to the station”*	8	6	14
**Total**				**126**	**92**	**218**
Consequence	9	Delay	*“I arrived late for my music class”*	3	0	3
		Detour	*“I had to go to the terminus and come back”*	18	11	29
		Difficulty reaching the destination	*“I never found the place; I was going round in circles”*	61	38	99
		Exploration	*“I discovered a beautiful district I wouldn’t have known otherwise”*	1	6	7
		Missed the desired means of transport	*“I arrived too late at the bus station”*	1	2	3
		Resulting harmful situation	*“I hurt my knee”*	5	0	5
		Resulting physical or emotional discomfort	*“It felt never-ending, I couldn’t take it anymore”*	14	11	25
		Simplification	*“I gained 15 min thanks to my friend”*	6	3	9
		Wait	*“We waited for 45 min”*	6	4	10
**Total**				**115**	**75**	**190**
Expressed emotion	7	Anger	*“It was very upsetting”*	26	25	51
		Fear	*“I was in utter panic”*	23	9	32
		Joy	*“I was very happy”*	0	9	9
		Neutral	*“I felt normal, I wasn’t stressed”*	13	12	25
		Sadness	*“I was sad”*	8	4	12
		Stress	*“It was stressful”*	14	20	34
		Other negative emotion	*“I was ashamed”*	13	9	22
**Total**				**97**	**88**	**185**
Problem-solving strategy	9	Changing to an alternative route	*“Eventually I got off the train earlier and walked”*	14	23	37
		Giving up	*“I thought “to hell with that” and I went home”*	3	1	4
		Going back	*“I walked back to find the right street”*	9	6	15
		Looking for a landmark	*“I looked up to find the bell tower”*	8	4	12
		None	*“I waited until the transit failure ended”*	24	16	40
		Planning	*“I used my app to see what to do”*	5	7	12
		Request for assistance	*“I called for them to pick me up”*	16	3	19
		Request for information	*“I asked some students my way”*	31	14	45
		Other	*“I tried to stay still and sat to avoid the heat”*	5	6	11
**Total**				**115**	**80**	**195**
Learning from the event	9	Would request assistance	*“I would call a cab”*	7	1	8
		Would request information	*“I would ask a bystander”*	3	1	4
		Would be more attentive	*“I would pay more attention”*	5	0	5
		Would manage by themselves	*“I wouldn’t ask for help and I’d look at the screen myself”*	3	1	4
		Would give up	*“I wouldn’t try to go there again”*	5	0	5
		Would not change anything	*“I would do the same”*	44	41	85
		Would plan	*“I would look at a map”*	9	21	30
		Would take another route	*“I would take the subway instead”*	4	5	9
		Other	*“I would do something else surely”*	7	6	12
**Total**				**87**	**76**	**163**

*Frequencies for each modality are retrieved from the analysis of the interviews.*

Among the variables, some could not be coded, resulting in 14.9% of missing data across the modalities. This missing information is labeled “N/A.”

### Statistical Analyses

#### Univariate Analyses

For each modality of the six variables, the number of occurrences was compiled into contingency tables, as detailed in Table 1. These frequencies were compared between the two groups using the two-sided Fisher’s exact test, as some frequencies are lower than five. Benjamini–Hochberg multiple comparison correction was applied to all data with a false discovery rate of 0.05 (Benjamini and Hochberg, 1995). An effect of the gender of the participants was also controlled for each variable, and did not seem to occur across all variables.

For each contingency table, we calculated Cramer’s V^2^, an estimator of the magnitude of the association between two categorical variables (Corroyer and Rouanet, 1994). Cramer’s V^2^ lies between 0 and 1. We considered the association as strong when V^2^ was greater than 0.16 and as weak when V^2^ was less than 0.04 (Wolff and Corroyer, 2004). We therefore analyzed the association when V^2^ was greater than 0.04.

In the case of significant statistical difference and V^2^ greater than 0.04, we calculated the association between each modality of the contingency table. Relative deviations (RDs) measure the associations and are determined on the basis of a comparison between observed and expected frequencies (i.e., those that would have been obtained if there was no association between the two variables) (Bernard, 2003). There is statistical attraction between two modalities when the RD value is positive, and statistical repulsion when it is negative. By convention, only RD with absolute terms greater than 0.25 are retained. All calculated RD are detailed in Table 2. When a modality occurred more than once but less than five times in total across the two groups, we ignored the strength of association between this modality and the groups of participants.

**TABLE 2:** Overview of the significant relative deviations (RD) between each modality of the critical incident variables and each group.

Variable	Modalities	Experimental group RD	Control group RD
Cause of the event			
Event type	Being in an unwanted or unusual route	–0.65	**0.90**
	Being lost	**0.28**	–0.39
	Conflict with another person	**0.56**	–0.76
	Disruption of the transportation network		**0.31**
	Harmful situation	**0.73**	–1.00
	Missing the transportation		
	Mistake by another person	–0.71	**0.97**
	Obstacle on the route	–0.53	**0.72**
	Physical or emotional discomfort		
	Route mistake		
	Transport not on time	**0.44**	–0.61
	Other		
Consequence			
Expressed emotion	Anger		
	Fear	**0.37**	–0.41
	Joy	–1.00	**1.10**
	Neutral		
	Sadness	**0.27**	–0.30
	Stress		
	Other negative emotion		
Problem-solving strategy	Changing to an alternative route	–0.36	**0.52**
	Giving up		
	Going back		
	Looking for a landmark		
	None		
	Planning	–0.29	**0.42**
	Request for assistance	**0.43**	–0.62
	Request for information		
	Other		**0.33**
Learning from the event	Would request assistance	**0.64**	–0.73
	Would request information		
	Would be more attentive	**0.87**	–0.1.00
	Would manage by themselves		
	Would give up	**0.87**	–1.00
	Would not change anything		
	Would plan	–0.44	**0.50**
	Would take another route		
	Other		

*Statistical attractions are in bold and repulsions in regular text. Non-significant RD are left blank. Variables that do not statistically differ between the two groups are left blank.*

#### Multiple Correspondence Analysis (MCA)

A multiple correspondence analysis (MCA) was performed in order to obtain a profile of the main types of existing complex situations and the group most associated with each one. We performed the MCA in accordance with the guidelines and recommendations provided by Le Roux and Rouanet (2010). This exploratory analysis determines the most significant associations between modalities across all selected variables by determining factorial axes that contribute to the overall variance.

Consistently with our objective of exploring the relationships between features of the complex situations as well as the projection of the group factor on them, we involved all the variables describing the complex events as active variables, while the “group” variable was added as a supplementary variable. Contrary to active variables, a supplementary variable does not contribute to the construction of the axes.

As positive emotions were not mentioned by the experimental group, no correspondence could be observed between the two groups for this emotion. We therefore did not include the “emotion” variable in this analysis. Also, as learning from the event consisted in a reflection after the situation rather than a factual description of the event itself, we excluded this variable from the analysis. We therefore performed the MCA on four active variables describing the characteristics of the situations (cause, type, consequence, problem-solving strategy).

Among the 218 incidents reported by the participants, incidents containing missing values (N/A) across the four selected active variables were excluded, since a missing value cannot correspond to any modality. Incidents containing “other” modalities were also excluded, since “other” covers heterogeneous unique modalities rather than a specific one. Overall positive incidents (corresponding to pleasant experiences or time saving situations) were excluded as a result of the absence of associated problem-solving strategies. In the end, the MCA was performed on 106 critical incidents (63 out of 126 from the experimental group, 43 out of 92 from the control group). In accordance with the requirements of this analysis, previously determined modalities for each variable were combined to reduce the number of modalities per active variable. These “broader” modalities used for the MCA are detailed in Table 3.

**TABLE 3:** Combined modalities obtained from the preliminary univariate analyses and used for the Multiple Correspondence Analysis.

Variable	Modality used for the MCA	Corresponding modalities combined from the preliminary univariate analyses
Cause of the event	Contextual	Choosing an unusual route
		Initial interindividual conflict
		Initial request for information
		Punctual environmental difficulty
	External	Environment legibility
		Transportation network
	Internal	Internal cause
		Not recognizing the environment
Event type	Mistake	Being lost
		Route mistake
	Obstruction	Mistake by another person
		Obstacle on the route
	Transport problem	Disruption of the transportation network
		Missing the transportation
		Transport not on time
	Unpleasant event	Being in an unwanted or unusual route
		Conflict with another person
		Harmful situation
		Physical or emotional discomfort
Consequence	Discomfort	Resulting harmful situation
		Resulting physical or emotional discomfort
		Wait
	Obstacle to achieving the goal	Difficulty reaching the destination
		Missed the desired means of transport
	Setback	Delay
		Detour
Problem-solving strategy	Autonomous action	Changing to an alternative route
		Going back
		Looking for a landmark
		Planning
	Passivity	Giving up
		None
	Asking someone for help	Request for assistance
		Request for information

The contribution of a modality to a factorial axis determines its coordinate on this axis, therefore allowing for a graphical representation of the MCA. The modalities that frequently appear together in the stories of the participants are graphically close to each other.

The interpretation of an axis is permitted by selecting the categories whose contributions exceeded the “baseline criterion,” which is determined by dividing 100 by the total number of active modalities included in the MCA. As detailed in Table 3, we included the 13 modalities of the incidents, therefore the baseline criterion we used equals 7.69%.

## Results

### Univariate Analyses: Describing the Complex Situations Step by Step in the Two Groups

#### Spatial Abilities and Events Recall

The Mann-Whitney test indicated that the control group recalled significantly more complex events (Median = 4) than the experimental group (Median = 3) (*U* = 247, *p* < 0.01). The events in the control group also appear to be more frequently judged positive than in the experimental group when comparing the two groups using a Chi-square test of independence [χ^2^(1) = 8.95, *p* < 0.01].

A *t*-test was performed on the six scores provided by the questionnaire on spatial abilities and did not indicate any significant difference between the two groups.

#### Differences in Event Types and Problem-Solving Strategies Between Groups

The types of events differed significantly between the two groups (*p* < 0.001). As a strong association between variables was found (V^2^ = 0.16), the associations between modalities (RD) were analyzed. There were four statistical attractions and three statistical repulsions in the experimental group. Compared to the control group, people with cognitive disabilities more frequently encountered situations centered on being lost, in conflict with another person, in a harmful situation or in a situation involving a transport schedule problem, either early or delayed. Less frequently than the control group, they found themselves on an unwanted or unusual route, suffered the consequences of a mistake made by another person, or met an obstacle on their route.

The problem-solving strategies implemented differed significantly between the two groups (*p* < 0.05). As a moderately strong association between variables was found (V^2^ = 0.09), the associations between modalities (RD) were analyzed. There was one statistical attraction and two statistical repulsions for the experimental group. Compared to the control group, people with cognitive disabilities more frequently chose to request assistance from another person, either a bystander or friend. Less frequently than the control group, they chose to change their current route for an alternative route, or stop to plan the rest of their trip. One statistical attraction for the control group was its relationship to the modality “other.”

Comparisons for the causes and consequences of the situations between the two groups returned no statistical significances. V^2^ and RD are therefore not discussed here.

#### Differences in Emotions and Learning Between Groups

The emotions generated by the events differed significantly between the two groups (*p* < 0.01). As the association between variables was moderately strong (V^2^ = 0.10), the associations between modalities (RD) were analyzed. There were two statistical attractions and one statistical repulsion for the experimental group. Compared to the control group, people with cognitive disabilities more frequently experienced emotions of fear and sadness when in a complex or unexpected situation. Less frequently than the control group, they experienced joy.

A comparison of the lessons learnt from the events by the two groups was statistically significant (*p* < 0.01). The association between variables was strong (V^2^ = 0.13), enabling the associations between modalities (RD) to be analyzed. There were three statistical attractions and one statistical repulsion for the experimental group. Compared to the control group, people with cognitive disabilities anticipated more frequently that if they were to encounter the same situation, they would request assistance, be more attentive or give up and not attempt the journey. Less frequently than the control group, they anticipated planning during the complex situation.

### Multivariate Analysis: The Main Profiles of Complex Situations

**FIGURE 1:**
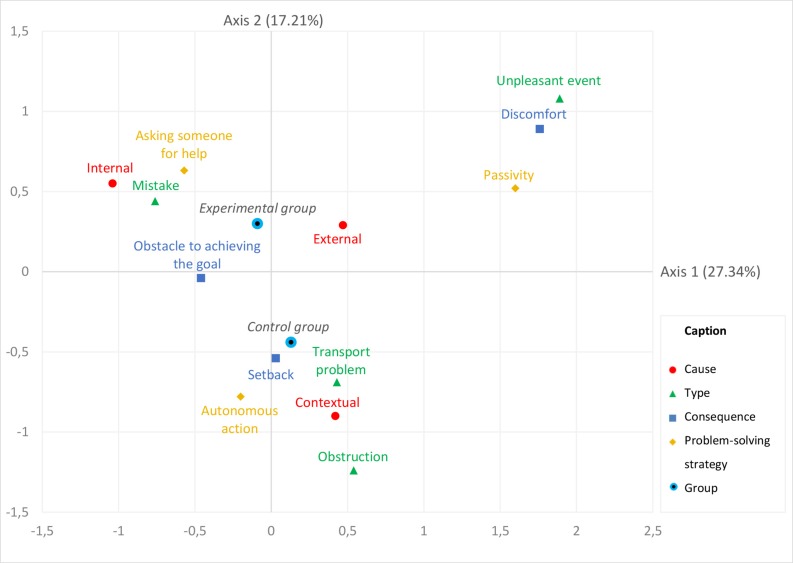
Graphical representation of the Multiple Correspondence Analysis. The coordinates of each modality are determined by its contributions to both axes. The modalities that frequently appear together in the stories of the participants are graphically close to one another. The contribution of each axis to the total variance is indicated in parentheses. Axis 1 (horizontal) opposes events relative to the individuals and events that arise in specific contexts and environments, independently of the individual. Axis 2 (vertical) opposes mainly the experimental and the control groups, with events relative to the control group being dealt with in a more autonomous manner and events relative to the experimental group relying more on the help of another person. The group variable, in italics, is used as a supplementary variable and therefore does not contribute to the overall variance.

Based on the decrease in the eigenvalues of the MCA, we considered the first two factorial axes for our analysis. They account for 44.55% of the total variance (axis 1 accounting for 27.34% and axis 2 for 17.21%). The contributions of each active modality are detailed in Table 4. The weight of the two modalities and the coordinates for the supplementary “group” variable are presented in Table 5. The graphical representation of the MCA is depicted in Figure 1.

**TABLE 4:** Contribution of each modality of the Multiple Correspondence Analysis to each axis; the columns “left,” “right,” or “top,” “bottom” refer to their coordinates.

Variable	Modality	Contribution to axis 1 (%)		Contribution to axis 2 (%)	
			
		Left	Right	Top	Bottom
Cause	Contextual		2.24		**16.15**
	External		3.48	2.15	
	Internal	**13.19**		5.86	
Type	Mistake	**11.68**		6.23	
	Obstruction		1.21		**10.38**
	Transport problem		2.24		**9.07**
	Unpleasant event		**15.1**	**7.83**	
Consequence	Discomfort		**20.17**	**8.25**	
	Obstacle to achieving the goal	5.44			0.07
	Setback		0.01		4.04
Problem-solving strategy	Autonomous action	0.69			**16.9**
	Passivity		**19.55**	3.27	
	Asking someone for help	4.99		**9.8**	
***Total***		***100%***		***100%***	

*The modalities whose contributions to an axis reach the baseline criterion to be used to interpret this axis are in bold.*

**TABLE 5:** Supplementary “group” variable’s weight and coordinates.

Modality	Weight	Coordinate on axis 1	Coordinate on axis 2
Control group	43	0.13	–0.44
Experimental group	63	–0.09	0.30

On the basis of the baseline criterion (7.69%) and the contribution of each modality, we used five modalities for the interpretation of axis 1 (“internal” cause, “mistake” and “unpleasant event” types, “discomfort” consequence, and “passivity” problem-solving strategy). Seven modalities were used for the interpretation of axis 2 (“contextual” cause, “obstruction,” “transport problem” and “unpleasant event” types, “discomfort” consequence, “autonomous action” and “asking someone for help” problem-solving strategies). Axis 1 opposes critical incidents relative to the individuals (“internal” cause, “mistake”) and critical incidents that arise independently from the individual (“unpleasant event,” “discomfort”), leaving a limited margin of maneuver to act on the situation (“passivity”). On the other hand, axis 2 opposes the critical incidents dealt with in an autonomous manner (“autonomous action”), and the critical incidents that require external intervention (“asking someone for help”).

To analyze the supplementary group variable, we used the deviation between the categories’ coordinates on the axes. A deviation between two categories greater than 0.5 is deemed notable (Le Roux and Rouanet, 2010). Axis 1 does not oppose the two groups, as the deviation between their coordinates on this axis is 0.22, as shown in Table 5. Axis 2 however opposes the two groups, as the deviation between their coordinates is 0.74. Thus, the experimental and control groups are mainly distinguished in regard to the way they deal with the complex situations, either by acting autonomously (for the control group) or asking for help (for the experimental group).

Overall, this MCA returned three main profiles of complex situations. The first profile we can identify is a complex situation resulting from an internal cause (e.g., “I did not pay attention”), which results in a mistake. In these situations, achieving the initial goal of the trip becomes uncertain. This situation is mainly encountered by people with a cognitive disability. A second situation deals with contextual problems, emerging because of particular circumstances mainly due to the action of other people (e.g., public works, transport network). This situation triggers autonomous problem-solving actions in order to resolve it. It is mainly encountered by the control group. Finally, a third type of situation is an unpleasant event, which causes discomfort and leaves the individual in a state of passivity (e.g., bad weather, crowded transportation). This situation is met mainly by the experimental group.

## Discussion

This study aimed to provide an overview of the difficulties actually experienced by people with cognitive disabilities regarding the complex situations they meet while getting around an urban area in their everyday lives. We used the critical incidents technique to identify the characteristics of the complex situations experienced by people with cognitive disabilities, and compared these characteristics to those of the situations encountered by matched controls. We took into account the situation itself as well as the actions implemented to solve a difficulty. Based on the semi-directed interviews, we divided a complex situation into different components, both factual (cause, event type, consequence and problem-solving strategy) and relative to an evaluation made by the participant (emotion triggered by the event, lessons learnt). We analyzed the potential differences between the two groups across these characteristics and determined a profile of the most frequently encountered complex situations. Based on our results, we propose recommendations for future navigational aids and further research on this matter.

### Complex Situations Experienced by People With Cognitive Disabilities Are Specific

While the causes of the encountered events appear to share similarities between the two groups, the types of situations encountered differ between people with and without cognitive disabilities. The complex situations met by control participants are mostly related to external events (unwanted route, disruption of the transportation network, having to deal with a mistake made by someone else or meeting a physical obstacle on the route). Conversely, while they also mention external events, participants with cognitive disabilities mostly describe themselves as the main protagonists of the complex situation. More frequently than the control group, they declare being lost and being in conflict with another person as representative complex events that happened to them.

In our study, being lost designates a situation in which the participant declares not knowing where to go anymore. We distinguished this situation from other events such as taking the wrong direction or being on an unwanted route. This representation of the situation of being lost therefore echoes the results of the study carried out by Sohlberg et al. (2005), which showed that people with cognitive disabilities avoid going outside for fear of getting lost. The present results confirm that being lost is indeed among the complex situations most frequently cited by people with cognitive disabilities. This finding is consistent with the result that taking an unwanted route, therefore running the risk of getting lost, seems to be overall avoided by people with cognitive disabilities in the first place: it is one of the least frequently mentioned events in the experimental group.

Another complex event frequently cited in the experimental group, but not by the control group, is the occurrence of interindividual conflict. These conflicts might thus be seen as a specificity of the experimental group and be related to the disability itself. As many professionals from the partner structures and participants themselves mentioned, the difficulty with cognitive disabilities lies in their absence of visibility. Consequently, other users as well as transport workers may behave with them exactly as with other people without taking their specific situation into account, which causes misunderstandings. Besides, it is well-documented that people with cognitive disabilities tend to experience social difficulties and mood disorders (for reviews, see Morton and Wehman, 1995; Carson et al., 2000), which could also increase the conflictual potential of a situation.

Interestingly, while they apparently mention similar causes of situations, the two groups mention different types of complex situations. A control participant may be more able to detect a potential change and adapt their route accordingly, therefore declaring not the initial precursor but the new route as problematic, whereas participants with a cognitive disability may experience difficulty in identifying and subsequently adapting their behavior to a new element that is not yet problematic.

### Consequences of the Complex Situations: Similarities Across Groups Suggesting Different Traveling Habits

The consequences of the complex situations did not differ between the two groups. Irrespective of the event type, people with and without cognitive disabilities have to make detours, end up being in uncomfortable situations (either physically or emotionally), or have to wait. This is not surprising, as among our sample of 218 critical incidents, 208 deal with situations where people have to go to a specific destination, often at a specific time. Uzan and Wagstaff (2018) proposed five possible categories of motives for an urban journey: physical activity, social activity (e.g., walking around with someone), exploration of an environment, regular route (e.g., going to work), or reaching a place, object or person. The latter two motives comprise more than 95% of the complex situations in our sample. It is worth noting that any consequence of a complex event during this type of journey might therefore disturb the traveler in their activity toward their goal, whatever the event is. These consequences would probably differ for perturbations occurring while doing physical activity outside, for example.

This observation suggests either that participants rarely encounter difficulties when going outside for other reasons than reaching a destination on time, or that they rarely go outside for the other three types of motives. The answer might actually be the latter for the experimental group: as Sohlberg et al. (2005) showed, people with cognitive disabilities avoid outside activities when possible. Therefore, it is not surprising that apart from the journeys that are mandatory (e.g., going to work, to an appointment), they avoid walking around or doing physical or social activity outside. Interestingly, this can be linked to the difference between the two groups regarding the number of complex situations mentioned: participants with cognitive disabilities recall fewer events than control participants. While this may be related to mnesic impairments, it could also be caused by the rarity of their journeys outside, therefore generating fewer complex events.

### Different Problems, Different Solutions: People With Cognitive Disabilities Ask for Help While Matched Controls Handle the Situation on Their Own

The problem-solving strategies implemented by the participants reveal an interesting potential for improvements, as they suggest a lesser degree of autonomy for the experimental group. A major distinction lies in the independence of the action taken: while control participants mostly change their route to an alternative one or plan a solution (either on their phone or on a physical map), the only statistical attraction for people with cognitive disabilities is directed toward the request of assistance from another person. This result therefore provides converging evidence with the findings of Lemoncello et al. (2010) who highlighted the use of the same problem-solving strategy in specifically designed situations where people had to follow incomplete instructions at crossroads. The present study is the first to encompass all the problem-solving strategies reported by people with cognitive disabilities in their everyday life. It is also the first study that compares strategies by people with cognitive disabilities with strategies in the control group when facing complex situations. From this comparison, it can be concluded that the request for assistance is the most frequently used strategy by people with cognitive disabilities. The MCA further strengthens this finding: taking into account all modalities across variables, the experimental group shows statistical ties with requesting help from another person, especially when the event is centered on a mistake (which, in our analysis, includes getting lost and mistaking the route). However, as Sohlberg et al. (2007) and Lemoncello et al. (2010) showed, individuals with cognitive disabilities tend to be vague or inaccurate in their request for help, as judged by experimenters as well as transport workers. This problem-solving strategy therefore appears to be ineffectual for this group.

Another interesting result lies in the statistical attraction of the controls toward the “other” types of problem-solving strategies. Across variables, the “other” modalities group unique events or actions that cannot be combined with any other modality. The attraction toward these “other” modalities in the problem-solving strategies could be interpreted as a form of opportunism, characterized by a greater diversity in the solutions implemented by the controls, as they appear to choose unique, unclassifiable solutions more frequently than people with cognitive disabilities.

### Subjective Experience of the Complex Situations: Negative Emotions and Reinforcement of the Strategies Already in Place for People With Cognitive Disabilities

The emotions triggered by complex events are unsurprisingly mostly negative for both groups. However, joy is mentioned in the control group only. In comparison, participants with a cognitive disability express mostly fear and sadness. This is congruent with what has been observed with the causes and event types: the negative emotions might be tied to the difficulty evaluating the origin of the situation and anticipating its evolution. Also, people with cognitive disabilities experience fewer situations that they judge positively overall, compared to the control group. While this evaluation of the event is the object of a separate question and is not necessarily related to the emotion actually triggered during the event, this difference between participants is consistent with the absence of joy observed in the experimental group. This finding is interesting from a wayfinding perspective, as it converges with existing evidence in the literature that emotion structures spatial representations (Storbeck and Maswood, 2016; Ruotolo et al., 2019). In particular, it has been reported that being in a positive mood and feeling positive emotions enhance spatial working memory by favoring a better retention of spatial information, in comparison to being in a negative mood (Storbeck and Maswood, 2016). Emotion has also been shown to affect spatial representations: participants who see landmarks inducing positive emotions while walking along a virtual route are able to locate the landmarks more accurately on a map afterward, as well as drawing the route, in comparison to participants who see landmarks inducing negative emotions (Ruotolo et al., 2019). These findings have led some researchers to advocate the use of positive emotion to improve wayfinding apps in everyday life, for example by computing instructions and routes based on street segments previously evaluated positively by users to allow for an “emotional” wayfinding (Gartner, 2012; Huang et al., 2014). Our results suggest that people with cognitive disabilities, who tend to experience mostly negative emotions when facing an unexpected situation, could also benefit from such a navigational aid proposing routes inducing positive emotions and therefore enhancing spatial working memory and “emotional” wayfinding.

The lessons learnt from the events confirm what is observed with the problem-solving strategies, as a difference emerges between the two groups. People with cognitive disabilities mention more frequently than the control group that they would request assistance, be more attentive or give up and not attempt to make the journey. This strengthens the previous finding that request for assistance seems to be a robust strategy for people with cognitive disabilities. Again, while they mention that they would privilege this action, they cannot plan the action in itself: they do not know in advance at which point of their trip or for what exact reason they would be in need of an outside person. This suggests that for this population, an assistive navigational aid should be available at all times to deal with losing their way at different locations.

The notion of being more attentive is an interesting finding, as it also strengthens the observation that complex situations may indeed emerge from an internal cause. Moreover, this is also congruent with the findings of Lemoncello et al. (2010): this anticipated change seems to be vague, as the participants cannot know in advance what it will be relevant to pay attention to. This also suggests that the directions provided by a navigational aid adapted to this population should tie directions to specific spatial landmarks in order to facilitate the focus of attention on elements that are relevant to the trip.

Giving up, which is also frequently mentioned by people with cognitive disabilities, further confirms the need for an improvement in the mobility of this population. This population, which already avoids most outside activities, contemplates giving up on journeys that are difficult, thereby increasing their difficulty in accessing leisure and social activities.

### Overall Remarks on the Complex Situations’ Profiles: Not All Events Call for a Wayfinding Improvement

The results of the MCA suggest that not all complex situations can be resolved with a navigational aid, as they do not systematically deal with the act of finding one’s way. A frequent profile of complex situations for people with cognitive disabilities concerns an unpleasant event, which causes discomfort, either physical or emotional (e.g., congested transportation, weather conditions). In these situations, the associated problem-solving strategy is mostly passivity, as people wait for the situation to end or simply follow the instructions given by transport workers. This complex situation profile does not seem to hint at a particular solution for people with cognitive disabilities in a wayfinding aid perspective, as they cope with unpleasant conditions rather than spatial cognition. However, while this profile of events does not tie in with decision making, orientation, path integration or closure (Vandenberg, 2016), it can directly impact the following of a path from an origin to a destination (Golledge, 1992). This is particularly true in the case of interindividual conflicts, an event type that occurs especially frequently for people with cognitive disabilities. Our results highlight the multifaceted nature of real-life wayfinding activities, which not only depend on the actual properties of the environment but also on non-spatial properties including the preferences, abilities and beliefs of an individual (Montello, 2017). This emphasizes the interest of taking into account mobility as a whole when discussing wayfinding for specific populations, as an event inducing an actual and recurring difficulty to reach a destination can arise separately from a disability involving the main components of Vandenberg’s (2016) model.

## Conclusion

This study examined the specific difficulties experienced by people with cognitive disabilities during wayfinding. Our perspective was exploratory. Our results show that people with cognitive disabilities encounter specific complex events and especially, that they get lost more frequently. Moreover, they rely more on the help of another person.

Some limitations of our study should be noted. First, as already mentioned, the pathogenesis heterogeneity among our experimental participants might weaken the generalizability of our results, and therefore calls for further studies on this matter. A second limitation directly concerns the cognitive disabilities themselves. Participants with a disability mention significantly fewer events than control participants, a difference that could be linked to the rarity of their urban journeys. Another possible explanation could be that participants with cognitive disabilities indeed suffer from memory impairments. These impairments could thus be a limitation for the validity of the data collected from our interviews.

Still, we can sketch out some recommendations for a navigational aid. As our results tie in with Vandenberg’s (2016) cognitive model of wayfinding on several levels, more specific suggestions toward an adapted navigational aid can be proposed. Vandenberg (2016) details four cognitive components of wayfinding: decision making, orientation, path integration, and closure. The analyses of the interviews highlight the relationships between these four components and two variables of complex situations: the event type and the problem-solving strategy implemented by the participant.

The event of “being lost” is among the most frequently encountered by people with cognitive disabilities, and can concern orientation, path integration and closure. One could argue that this situation is already taken into account by existing navigational aids. However, to solve such complex situations, most of the existing solutions provide exclusively bird’s-eye views and information (Siegel and White, 1975) which do not meet human needs well as they deal with the most complex levels of spatial representations (Golledge, 1991; Grison and Gyselinck, 2019). These solutions might therefore not be sufficiently helpful for people with cognitive disabilities, since they encounter difficulties mostly based on orientation, path integration or closure when they get lost. The provision of less complex spatial information, such as that based on landmarks, could therefore be considered as more adapted to help this population. Moreover, as “being lost” also refers to situations where individuals do not recognize their destination, obstructing the “closure” part of wayfinding, the analyses of the interviews suggest that an adapted aid should also be able to ease this last step of the journey by describing the destination, either verbally or by showing a picture, in order to make it recognizable by the user.

The problem-solving strategy variable can be directly tied in with the decision-making part of wayfinding. The finding that the most frequently used problem-solving strategy by people with cognitive disabilities is to ask another person for assistance especially hints at a potential improvement for these navigational aids. While people with cognitive disabilities ask someone for help more often than they implement any other strategy, and especially when they are facing an obstacle to the goal of their journey, it has been documented that their requests are often vague, making it difficult for the helper to understand the need and provide sufficient help (Sohlberg et al., 2007; Lemoncello et al., 2010). Still, a possible explanation for the high frequency of use of this strategy despite its flaws lies in the fact that when prompted to describe a route, most people do not use bird’s-eye view information such as current aids or maps: they rely on landmarks, which are considered as the key components of route descriptions (Denis, 1997). More specifically, people associate an action to a landmark in order to give directions (Denis et al., 2007). This landmark-based level of spatial information may explain the interest of people with cognitive disabilities in this strategy, also indicated by their intention to ask for help again in the future, as shown by the analysis of the variable “learning from the event.” Then again, one could argue that most navigational aids already provide vocal features that could replace information provided by a bystander. However, in current systems, the content of vocal instructions also differs from what a person would actually give. Therefore, an adapted navigational aid should aim at better matching the directions a real person would give and provide instructions linking a landmark to the action to be performed. This would make the aid more relevant to the needs and actual problem-solving strategies of people with cognitive disabilities. Finally, in addition to landmark-based information, considering the negative subjective experience and emotions felt by people with cognitive disabilities, our results are in favor of the use of positive emotions-inducing landmarks in adapted navigational aids, as recommended by several authors, to support a better memorization of spatial information (Gartner, 2012; Huang et al., 2014; Ruotolo et al., 2019).

This study also suggests perspectives for future research. The analysis of the answers to the questionnaire on spatial abilities (Pazzaglia et al., 2000) does not indicate any difference in general spatial orientation, suggesting both people with and without cognitive disabilities have similar spatial skills. Yet, as has been documented, a cognitive disability can be related to several impairments in spatial representations and wayfinding (Lemoncello et al., 2010; Claessen and van der Ham, 2017). Our results provide evidence that people with cognitive disabilities get lost more often than controls. This absence of difference in the questionnaire on spatial abilities therefore does not substantiate the literature, in which most studies have focused only on participants facing prior difficulties in wayfinding. While the present study might indicate an inadequacy of the questionnaire for the target population, or a failure of the questionnaire to detect a difference between people with cognitive disabilities and matched controls, other results suggest a more nuanced picture. Claessen et al. (2017) carried out a study on people with documented cognitive disabilities resulting from strokes. Among their sample of 77 participants, only 33 (43%) actually mentioned difficulties in wayfinding. Moreover, among these 33 people, seven did not show any impairment in internal spatial representations when compared to matched controls over cognitive tests. These data suggest that among the target population, some people do not experience difficulties in wayfinding, and some do not experience difficulties in internal spatial representations. Importantly, these sub-populations may not entirely overlap. Therefore, we cannot rule out that the difficulties observed in wayfinding in the present study do not translate into differences in auto-evaluation of general spatial abilities as measured by the questionnaire. This strengthens the need to supplement quantitative measures by qualitative investigations, allowing deeper understanding of all the dimensions implied in the diverse wayfinding situations encountered by individuals.

## Data Availability Statement

The datasets generated for this study are available on request to the corresponding author.

## Ethics Statement

Ethical review and approval was not required for the study on human participants in accordance with the local legislation and institutional requirements. The patients/participants provided their written informed consent to participate in this study.

## Author Contributions

RD designed the experiment, recruited subjects, acquired, analyzed and interpreted the data and drafted the manuscript. J-MB designed the experiment, substantially contributed to the interpretation of the data and critically revised the manuscript. VG designed the experiment, substantially contributed to the interpretation of the data and critically revised the manuscript. All authors gave final approval for publication and agreed to be accountable for all aspects of the work in ensuring that questions related to the accuracy or integrity of any part of the work are appropriately investigated and resolved.

## Conflict of Interest

The authors declare that the research was conducted in the absence of any commercial or financial relationships that could be construed as a potential conflict of interest.
